# Identification of Koumine as a Translocator Protein 18 kDa Positive Allosteric Modulator for the Treatment of Inflammatory and Neuropathic Pain

**DOI:** 10.3389/fphar.2021.692917

**Published:** 2021-06-24

**Authors:** Bojun Xiong, Guilin Jin, Ying Xu, Wenbing You, Yufei Luo, Menghan Fang, Bing Chen, Huihui Huang, Jian Yang, Xu Lin, Changxi Yu

**Affiliations:** ^1^ Department of Pharmacology, School of Pharmacy, Fujian Medical University, Fuzhou, China; ^2^ Key Laboratory of Gastrointestinal Cancer (Fujian Medical University), Ministry of Education, School of Basic Medical Sciences, Fujian Medical University, Fuzhou, China; ^3^ Fujian Key Laboratory of Drug Target Discovery and Structural and Functional Research, School of Pharmacy, Fujian Medical University, Fuzhou, China

**Keywords:** koumine, TSPO, positive allosteric modulation, inflammatory pain, neuropathic pain

## Abstract

Koumine is an alkaloid that displays notable activity against inflammatory and neuropathic pain, but its therapeutic target and molecular mechanism still need further study. Translocator protein 18 kDa (TSPO) is a vital therapeutic target for pain treatment, and recent research implies that there may be allostery in TSPO. Our previous competitive binding assay hint that koumine may function as a TSPO positive allosteric modulator (PAM). Here, for the first time, we report the pharmacological characterization of koumine as a TSPO PAM. The results imply that koumine might be a high-affinity ligand of TSPO and that it likely acts as a PAM since it could delay the dissociation of ^3^H-PK11195 from TSPO. Importantly, the allostery was retained *in vivo*, as koumine augmented Ro5-4864-mediated analgesic and anti-inflammatory effects in several acute and chronic inflammatory and neuropathic pain models. Moreover, the positive allosteric modulatory effect of koumine on TSPO was further demonstrated in cell proliferation assays in T98G human glioblastoma cells. In summary, we have identified and characterized koumine as a TSPO PAM for the treatment of inflammatory and neuropathic pain. Our data lay a solid foundation for the use of the clinical candidate koumine to treat inflammatory and neuropathic pain, further demonstrate the allostery in TSPO, and provide the first proof of principle that TSPO PAM may be a novel avenue for the discovery of analgesics.

## Introduction

Koumine is the most abundant monomer compound in the crude alkaloid extract of *Gelsemium elegans*. Previous clinical evidence has shown that the parenteral solution of crude *Gelsemium* alkaloid extraction has potent analgesic effects (Chen, 1984). However, the further clinical use of the crude alkaloid extract has been limited by its narrow therapeutic index. Koumine, an alkaloid with low toxicity and a high therapeutic index, displays notable activities against inflammatory and neuropathic pain, with no tolerance or dependence (Xu et al., 2012; Ling et al., 2014; Yang et al., 2016; Xiong et al., 2017; Jin et al., 2018a; Jin et al., 2018b; Jin et al., 2019). A series of studies by our research group showed that koumine has significant value in new drug development (Yu et al., 2015), but the therapeutic target and molecular mechanism still need further study.

The translocator protein 18 kDa (TSPO) is a 5-transmembrane domain protein that is localized primarily in the outer mitochondrial membrane and is expressed in most tissues examined (Papadopoulos et al., 2006; Papadopoulos et al., 2017). TSPO is involved in cell proliferation and steroid hormone synthesis, and TSPO activation is reported to be beneficial for several kinds of neurological diseases, such as inflammatory and neuropathic pain (DalBo et al., 2004; Hernstadt et al., 2009; Wei et al., 2013; Liu et al., 2016). Multiple ligand binding sites exist on TSPO, and several classes of ligands have been identified, such as TSPO endogenous ligand protoporphyrin IX, and the typical exogenous ligands PK11195 and Ro5-4864 (Rao and Butterworth, 1997; Li and Papadopoulos, 1998; Li et al., 2015). Although recent studies involving the genetic depletion of TSPO have raised controversy about the functions of TSPO (Banati et al., 2014; Morohaku et al., 2014; Tu et al., 2014; Fan et al., 2015; Tu et al., 2015; Chung et al., 2020), the evolutionary conservation of TSPO from bacteria to humans, which has been traced to 3.5 billion years ago, indicates that it is still a vital target that deserves further exploration (Fan et al., 2012).

Given that TSPO carries multiple spatially distinct ligand binding sites, allostery may occur in TSPO. Allostery is a universal phenomenon whereby a perturbation by an effector at one site of the molecule leads to a functional change at another (Nussinov and Tsai, 2013). It is regarded as “the second secret of life,” and is a key biological phenomenon for understanding biological systems (Fenton, 2008). Compared with traditional orthosteric ligands, allosteric modulators showed higher receptor selectivity and can maintain the temporal and spatial activity of receptor signals. Therefore, allosteric modulators have the advantages of high selectivity, strong safety and no tolerance. These advantages have prompted allostery to become a new direction for drug development and several efficient allosteric modulators have been approved as marketed drugs (Wootten et al., 2013). In recent years, researchers have explored the allostery in TSPO from the perspectives of cell function, ligand dissociation kinetics and structure (Narlawar et al., 2015; Jaipuria et al., 2017; Rojas et al., 2018), but further investigation is still needed. Our previous competitive binding assay have shown that instead of an competitive inhibition, koumine significantly increased the binding of ^3^H-PK11195 with TSPO from the rat cerebral cortex (Jin, 2014), a hallmark of a positive allosteric modulator (PAM) (Christopoulos et al., 2014). Therefore, systematic research is needed.

In this study, we used a multidisciplinary approach to characterize koumine as a TSPO PAM, and examined whether the allostery has therapeutic effects *in vivo*. First, reliable and label-free surface plasmon resonance (SPR) technology was adopted to evaluate the affinity between koumine and TSPO. Then, the effect of koumine on the dissociation of ^3^H-PK11195 from TSPO from rat cerebral cortex was tested. As converging lines of evidence imply a vital role for TSPO in inflammatory and neuropathic pain, Ro5-4864, a representative TSPO ligand, mediated analgesic and anti-inflammatory effects were tested in the absence or presence of koumine in several acute and chronic inflammatory and neuropathic pain models. The possible positive allosteric modulatory effect of koumine was further explored in two cell functional assays: inhibition of T98G human glioblastoma cell proliferation and stimulation of C6 cell pregnenolone production.

## Materials and Methods

### Chemicals and Reagents

Koumine was isolated from *Gelsemium elegans* Benth. using our previously established method of pH-zone-refining counter-current chromatography, which is capable of obtaining a purity of 99% (Su et al., 2011). ^3^H-PK11195 (specific activity, 85.1 *μ*Ci nmol^−1^) was purchased from PerkinElmer Life Sciences. PK11195 and Ro5-4864 were purchased from Sigma-Aldrich. Lipids were purchased from Avanti Polar Lipids. SPR experiments were performed on a Biacore T200 system using series S L1 and S CM5 sensor chips (GE Healthcare).

### Animals

Male ICR mice, male Lewis rats and male SD rats (purchased from Beijing Vital River Laboratory Animal Technology Co., Ltd.) weighing 18–22, 130–150 and 130–150 g, respectively, were used. The animals were group-housed, and the room was kept at 24 ± 2°C and 50–60% humidity under a 12/12 h light/dark cycle. The animals had *ad libitum* access to food and water. All experimental procedures were approved by the Committee of Ethics of Fujian Medical University, China, and all applicable institutional and/or national guidelines for the care and use of animals were followed (Zimmermann, 1983).

### Surface Plasmon Resonance Experiments

Cells of the BL21(DE3) pLysS *Escherichia coli* strain were transformed with the pET15b vector, from which the expression of mouse TSPO recombinant protein was induced by 1 mM isopropyl-1-thiol-*β*-D-galactopyranoside as previously described (Robert and Lacapère, 2010; Senicourt et al., 2017). Cells were harvested and resuspended in lysis buffer (150 mM NaCl and 50 mM HEPES-Na, pH 7.8) with the addition of 1 mg/ml lysozyme and sonicated thoroughly. The lysate was centrifuged at 15,000 g at 4°C for 20 min to pellet the insoluble inclusion bodies. The final pellet of inclusion bodies was solubilized in binding buffer (150 mM NaCl, 50 mM HEPES-Na, 1% SDS (wt/vol), pH 7.8) and was purified by the Ni-NTA gravity flow column.

Proteoliposomes composed of DMPC/DMPE (9:1) and TSPO in a 5:1 (wt/wt) ratio were prepared using a previously described method (Ostuni et al., 2010). Liposomes composed of DMPC/DMPE (9:1) were prepared as published previously (Hatty et al., 2014). A few milligrams of DMPC and DMPE were weighed into a round-bottom glass flask, and chloroform and methanol were added to dissolve them. A thin lipid film was prepared by a rotary evaporator and dried under vacuum for at least 3 h to remove all traces of chloroform and methanol. The dry lipid film was resuspended in PBS buffer (10 mM phosphate buffer, 2.7 mM KCl, 137 mM NaCl) to a concentration of 1 mg/ml, hydrated for 2 h at 45–55°C, and finally sonicated to produce small unilamellar vesicles. The suspension of small unilamellar vesicles was then kept at 45°C for use on the same day. The sizes of proteoliposomes and liposomes were determined by dynamic light scattering using Nicomp 380 Z3000 instrument (Parficle Sizing Systems, America). The results show that the size of proteoliposomes and liposomes were 334.7 ± 13.9 and 353.3 ± 10.3 nm (Supplementary Figure S1), respectively.

SPR analyses were performed at 25°C with a Biacore T200 instrument. For proteoliposomes and liposomes (reference), they were immobilized on a series S L1 sensor chips. It is possible that the repeated regeneration steps in the multicycle kinetics process impairs the activity of TSPO proteoliposomes, thus we were unable to use multicycle kinetics to effectively determine the affinity of TSPO and its ligands. Therefore, the single cycle kinetics, which assumes to be as robust as the classical multicycle kinetics for measuring affinity, was used. Kinetic rate constants for the various analytes were determined using single cycle protocols where five serial 2-fold dilutions of the analytes were injected for 180 s without intervening regeneration, followed by a 300 s dissociation phase after the last injection. Interactions were measured at 30 *μ*l/min. PBS, with addition of 1% DMSO (pH 7.4), was used as running buffer. Three consecutive 1 min injections with 10 *μ*l/min of glycine HCl 3.0 at the end of each single-cycle kinetics regenerated the chip. For mouse TSPO in a lipid-free environment, affinity was measured according to established methods with minor modifications (Zhang et al., 2015). Briefly, HBS-EP buffer was used as the running buffer. The mouse TSPO recombinant protein was captured on a series S CM5 sensor chip *via* an anti-His antibody (His capture kit, GE healthcare). Next, serial dilutions of the analytes in running buffer were injected over the mouse TSPO recombinant protein. After each sample injection, the surface was regenerated with 0.75 mM NaOH solution for 30 s at a rate of 20 *μ*l/min. Kinetic parameters were derived from data sets acquired in multicycle kinetics. Sensorgrams show blank and reference subtracted data, and a DMSO correction was also applied. With the Biacore T200 Evaluation 2.0 software, we globally fitted the double blank-referenced data by nonlinear regression to a Langmuir 1:1 interaction model.

### Radioligand Binding Assays

^3^H-PK11195 binding assays were performed with the method previously described (Benavides et al., 1983; Awad and Gavish, 1987). Briefly, male SD rats (240 ± 10 g) were killed by decapitation, and the cerebral cortices were dissected. The tissues were homogenized in 10 volumes (ml/g) of 50 mM Tris HCl buffer containing 0.32 M sucrose (pH 7.4). The homogenates were centrifuged at 1,000 g for 10 min at 4°C. The resulting pellets were discarded, and the supernatants were centrifuged at 9,000 g for 15 min. The pellets were resuspended in 10 volumes of Tris HCl buffer containing 0.32 M sucrose (pH 7.4) and centrifuged at 13,000 g for 30 min, then resuspended in 10 volumes of Tris HCl buffer and centrifuged again at 13,000 g for 30 min. The final pellets were suspended in Tris HCl buffer and used in the binding assays. Protein concentrations were measured using the BCA Protein Assay Kit (ThermoFisher Scientific) according to the manufacturer’s instructions.

In the saturation binding experiments, incubations were initiated by the addition of the cerebral cortices (50 *μ*g/tube) to a final volume of 500 *μ*l of 50 mM Tris HCl buffer (pH 7.4) containing 0.125–32 nM ^3^H-PK11195. After incubating at 4°C for 2 h, the reaction was terminated by the addition of ice-cold 50 mM Tris HCl buffer (pH 7.4) followed by vacuum filtration through Filtermat A glass microfiber filters (pretreated with 0.2% polyethylenimine) using a Brandel cell harvester. The filters were washed rapidly, and radioactivity was determined by liquid scintillation spectrometry. Specific binding is defined as the difference between total binding and nonspecific binding in the presence of 1000-fold excess unlabeled PK11195.

In the equilibrium binding assays, incubations were initiated by the addition of the cerebral cortices (50 *μ*g/tube) to a final volume of 500 *μ*l of 50 mM Tris HCl buffer (pH 7.4) containing 0.1 nM (∼0.2 K_D_) ^3^H-PK11195 and 10^−12^–10^−3^ M koumine or vehicle. After incubation at 4°C for 2 h, the reaction was terminated by the addition of ice-cold 50 mM Tris HCl buffer (pH 7.4) followed by vacuum filtration through Filtermat A glass microfiber filters (pretreated with 0.2% polyethylenimine) using a Brandel cell harvester. The filters were washed rapidly, and radioactivity was determined by liquid scintillation spectrometry. Specific binding is defined as the difference between total binding and nonspecific binding in the presence of 1000-fold excess unlabeled PK11195.

In the dissociation kinetics experiment, the cerebral cortices (50 *μ*g/tube) were first incubated with 10 nM ^3^H-PK11195 at 4°C for 2 h to achieve pre-equilibration, and 10^−10^ M, 10^−6^ M (final concentration) or vehicle was then added to the reaction buffer, followed by the addition of PK11195 (10 *μ*M) to initiate the dissociation process. Then, samples were filtered through Filtermat A glass microfiber filters (pretreated with 0.2% polyethylenimine) using a Brandel cell harvester, and the radioactivities were measured at the indicated time points.

### Formalin Test

The formalin test was assessed as described by Dubuisson and Dennis (Dubuisson and Dennis, 1977) with minor modifications. Briefly, mice were allowed to acclimate for at least 30 min in the transparent plastic box before formalin injection. Each animal was injected with 10 *μ*l of 5% formalin into the dorsal surface of the right hindpaw. Mice were then observed at 0–5 min (phase one) and at 11–60 min (phase two) after formalin, and the amount of time spent licking or biting the injected paw was recorded as the nociceptive response. Ro5-4864 or vehicle (5% DMSO) was administered s. c. 30 min prior to the formalin injection, and koumine was administered s. c. 40 min before formalin injection. To determine whether koumine potentiated the anti-inflammatory pain effects of Ro5-4864, koumine or its vehicle was injected 10 min before Ro5-4864 injection. Ro5-4864 was dissolved in DMSO and diluted in sterile physiological saline, and the final concentration of DMSO was 5%. Koumine was dissolved in sterile physiological saline directly. Animals were randomized for treatment and behavioral test were performed in a blinded manner.

### Collagen-Induced Arthritis Model

The induction of CIA in Lewis rats followed protocols and used reagents supplied by Chondrex. Bovine type II collagen (2 mg/ml in 0.05 M acetic acid) was emulsified with incomplete Freund’s adjuvant (IFA; Sigma-Aldrich) using an electric homogenizer equipped with a small blade. Equal volumes of collagen and IFA were mixed in an ice water bath, and collagen was added dropwise to the IFA at 1,000–3,000 rpm. The emulsion was ready when it became a solid clump that did not dissipate when dropped in water. Lewis rats were anesthetized and received 0.2 ml of collagen emulsion by subcutaneous injection at the base of the tail. Booster injections were given on day 7 of the study with 0.1 ml of emulsion. Immunized rats received Ro5-4864, koumine or vehicle for seven consecutive days beginning from day 29 after the first collagen injection. The mechanical withdrawal threshold (MWT) was determined on day 29 and day 35, the hind paw volume was determined on day 35, and was measured 60 min following the MWT measurement. Koumine or vehicle was injected s. c., and MWT values were determined 60 min after koumine injection. Ro5-4864 or vehicle was injected i. p., and MWT was determined 50 min after Ro5-4864 injection. To determine whether koumine potentiated the effects of Ro5-4864, koumine (1.0 mg/kg, s. c.) or vehicle was injected 10 min before Ro5-4864 (0.0625–1.0 mg/kg, i. p.) injection, and the MWT was determined 50 min after the last injection. Animals were randomized for treatment and behavioral test were performed in a blinded manner. Mechanical allodynia and edema measurement were measured using the procedures described below. Only rats with mechanical withdrawal threshold scores (calculated by mechanical withdrawal threshold of immunized rats/mechanical withdrawal threshold of normal rats) on day 28 after the first collagen injection >0.75 were used in the study. Moreover, animals demonstrating motor deficit were excluded (Yang et al., 2016).

### Chronic Constriction Injury Model

The rat CCI model of neuropathic pain was produced according to the method described by Bennett and Xie (Bennett and Xie, 1988). Adult male SD rats were anesthetized by isoflurane delivered by using the anesthetic machine. The right common sciatic nerve was isolated at the mid-thigh level and loosely ligated using four chromic gut (4–0) ties separated by intervals of 1 mm. For sham surgeries, the right sciatic nerve was exposed using the same procedure, but the nerve was not ligated. On the 9th day after surgery, Ro5-4864 or vehicle (5% DMSO) was administered i. p. 50 min before the measurement of mechanical allodynia, and koumine was administered s. c. 60 min before the measurement of mechanical allodynia. To determine whether koumine potentiated the analgesic effects of Ro5-4864, koumine (0.28 mg/kg, s. c.) or vehicle was injected 10 min before Ro5-4864 (0.0625–1.0 mg/kg, i. p.) injection on day 9 after CCI surgery, and the MWT was determined 50 min after the last injection. Animals were randomized for treatment and behavioral test were performed in a blinded manner. Mechanical allodynia was measured using the procedures described below. Only rats with mechanical withdrawal threshold scores (calculated by CCI ipsilateral paw mechanical withdrawal threshold/contralateral paw mechanical withdrawal threshold) on day 8 after CCI surgery >0.75 were used in the study. Moreover, animals demonstrating motor deficit were excluded (Xu et al., 2012).

### Measurement of Mechanical Allodynia

Mechanical allodynia was measured using a commercially available electronic von Frey apparatus (Model 2,390; IITC Life Science Inc., Woodland Hills, CA) as described by Mitrirattanakul (Mitrirattanakul et al., 2006) with minor modifications. Rats were placed in a Plexiglas box on a steel mesh floor, and analyses were performed using an electronic von Frey apparatus. Stimulation was applied to the center of the hind paw in an upward motion of the von Frey filament until foot withdrawal occurred, and the withdrawal threshold was automatically recorded. The maximum strength of the filament used for von Frey testing was 55 g. The procedure was repeated three times at approximately 5 min intervals for each hind paw, and the MWT was calculated as the mean of the three latencies.

### Edema Measurement

Arthritic edema was assessed by measuring the hind paw volume with a plethysmometer (Yi Yan Technology Development Co., Ltd.) according to the manufacturer’s recommendations.

### Cell Culture

Rat C6 glioma cells (National Infrastructure of Cell Line Resource, China) and T98G human glioblastoma cells (a kind gift of professor Zucheng Ye, Fujian Medical University, China) were cultured in high-glucose DMEM supplemented with 10% fetal bovine serum (Gibco) and 1% penicillin and streptomycin. Cultures were maintained in a humidified atmosphere of 5% CO_2_ at 37°C.

### Cell Proliferation Assay

T98G cells were plated at 1 × 10^4^ cells/well in 96-well plates. 24 h later, cells were treated with koumine, PK11195 or Ro5-4864, as well as a vehicle control (0.5% DMSO). After 48 h of drug exposure, proliferation was indexed using a BrdU ELISA Kit (Roche Molecular Diagnostics) according to the manufacturer’s protocol, and absorption was measured at 370 nm (reference wavelength 492 nm).

### Cell Viability Assay

T98G cells were plated and treated as in the proliferation assays. After 48 h of drug exposure, CellTiter Blue Reagent (Promega) was added to the cells and incubated for 4 h in 5% CO_2_ at 37°C. Absorption was measured at 570 nm using 600 nm as a reference wavelength.

### Pregnenolone Measurement

Pregnenolone assessment was performed using rat C6 glioma cells as previously described (Papadopoulos et al., 1992; Karlstetter et al., 2014; Costa et al., 2016). C6 cells were plated at 1 × 10^4^ cells/well in 96-well plates. 24 h later, the cells were washed 2 times with salt medium consisting of 140 mM NaCl, 5 mM KCl, 1.8 mM CaCl_2_, 1 mM MgSO_4_, 10 mM glucose, and 10 mM HEPES-NaOH (pH 7.4) plus 0.1% bovine serum albumin. For the measurement of pregnenolone secreted into the medium, the further metabolism of pregnenolone was blocked by the addition of 25 *μ*M trilostane to the salt medium. The addition of koumine, PK11195 or Ro5-4864 to C6 cells was accomplished by a complete change of the salt medium to a medium containing the appropriate drug concentration. The final concentration of DMSO was constant for all the wells within each experiment and did not exceed 0.5% (v/v), a concentration that on its own had no effect on steroid production. At the end of the incubation periods (2 h), the cell medium was collected, and the amount of pregnenolone secreted into the medium was quantified by a pregnenolone ELISA kit according to the manufacturer’s recommendations (IBL International). Absorption was measured at 450 nm.

### Data and Statistical Analysis

Concentration response data were fitted by Graphpad Prism Version 5.01 (GraphPad Software Inc.) to provide estimates of EC_20_, EC_50_ and E_max_. Saturation binding data were fitted to one-site saturation curve and dissociation kinetic data in the absence or presence of koumine were fitted to a mono-exponential function, the data processing method is consistent with the literature (Guo et al., 2013; Korczynska et al., 2018). The data of behavioral tests were analyzed using the independent samples *t*-test and when appropriate with one-way ANOVA followed by the LSD post hoc test. Differences were considered statistically significant when *p* < 0.05, statistical analyses were performed with SPSS (version 19.0, SPSS Inc.). The data and statistical analysis comply with the recommendations on experimental design and analysis in pharmacology (Curtis et al., 2018).

## Results

### Koumine is a High-Affinity Ligand of Translocator Protein

**FIGURE 1:**
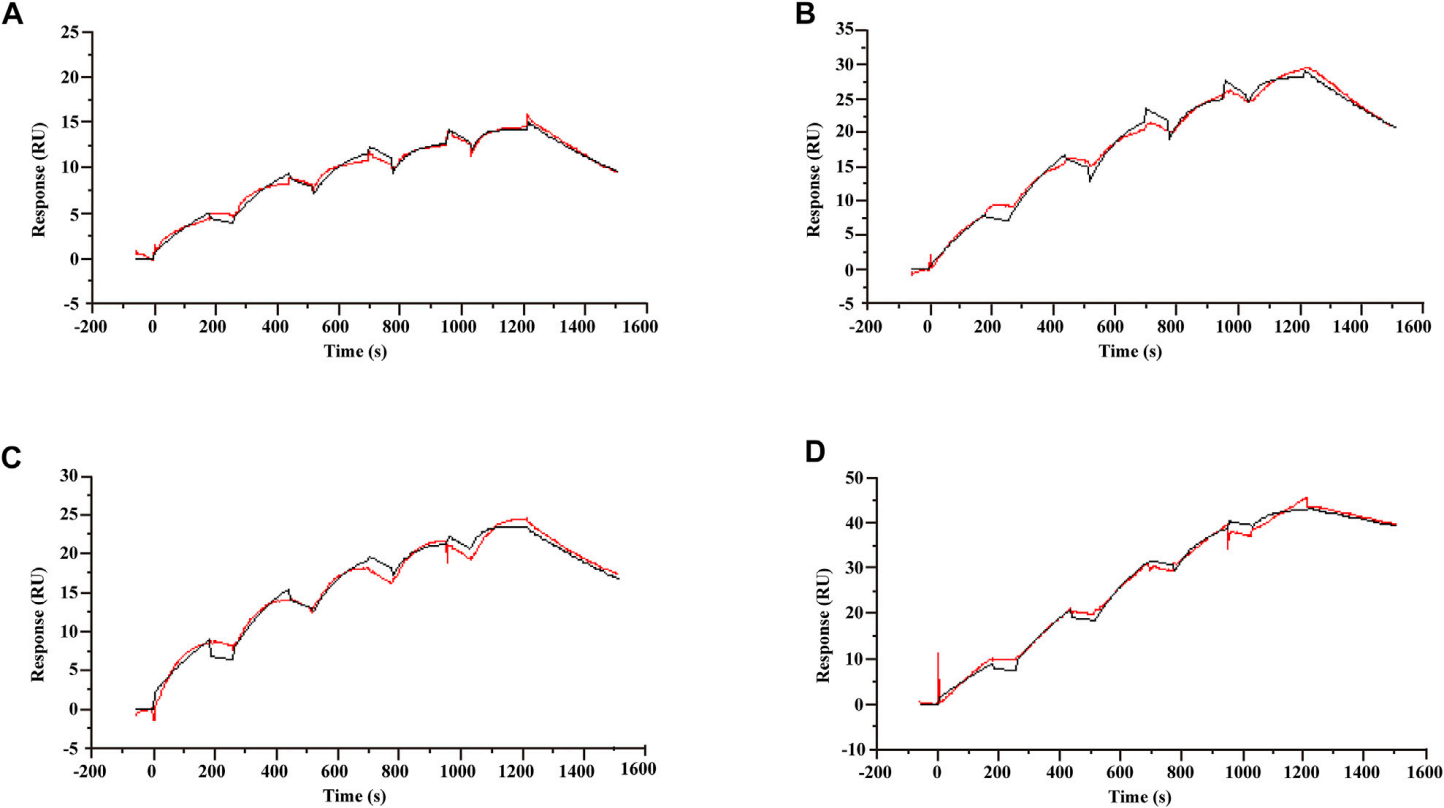
Kinetic profile for Ro5-4864, PK11195, protoporphyrin IX and koumine binding to TSPO proteoliposomes determined using single-cycle kinetics. **(A–D)** The sensorgram of Ro5-4864 **(A)**, PK11195 **(B)**, protoporphyrin IX **(C)** and koumine **(D)** binding to the TSPO proteoliposomes. The Ro5-4864, PK11195 and koumine concentrations were 3.125, 6.25, 12.5, 25 and 50 nM, and the protoporphyrin IX concentrations were 25, 50, 100, 200 and 400 nM. The running buffer contained 10 mM phosphate buffer, 2.7 mM KCl, 137 mM NaCl and 1% DMSO. Binding data were collected at a flow rate of 30 μl/min. The association and dissociation phases were 180 and 300 s, respectively. The red traces represent the experimental data, and the black traces represent the global fit of the data to a Langmuir 1:1 interaction model. Sensorgrams show blank and reference subtracted data, and a DMSO correction was also applied. A representative result from three independent experiments is presented.

A characteristic of allosteric modulators is their ability to bind receptors and modulate the binding and/or signaling efficacy of orthosteric ligands (Wootten et al., 2013). To validate that koumine can bind TSPO directly, we used SPR, a label-free, real-time technology to determine biomolecular interactions. After TSPO proteoliposomes and TSPO-free liposomes were captured on series S L1 sensor chip, a series of concentrations of ligand were injected. Single-cycle kinetics were used to analyze the binding kinetics. Consistent with the affinity determined in radioligand binding assays (Awad and Gavish, 1987; Verma et al., 1987), the K_D_ values of Ro5-4864, PK11195 and protoporphyrin IX were 2.06 ± 0.11, 1.97 ± 0.02 and 12.62 ± 0.39 nM (Figures 1A–C), respectively. By following the above setup, we determined the affinity of koumine with TSPO. As shown in Figure 1D, the K_D_ of koumine was 0.86 ± 0.07 nM, which was significantly lower than the values of Ro5-4864, PK11195 or protoporphyrin IX (*p* < 0.05 for Ro5-4864 and PK11195, *p* < 0.001 for protoporphyrin IX), indicating that koumine is a high-affinity ligand of TSPO.

We have also noticed that PK11195 can also interact with lipid bilayers and the heterogeneity of TSPO protein orientation in proteoliposomes (Hatty et al., 2014), which may complicate the determination of TSPO and ligand affinity. Therefore, we studied the affinity of mouse TSPO with related ligands in a lipid-free environment. The results showed that the affinity of Ro5-4864, PK11195 and koumine to mouse TSPO was 169.75 ± 6.07, 161.33 ± 6.1 and 97.37 ± 1.76 *μ*M (Supplementary Figure S2), respectively. These results support our assumption that koumine may bind to TSPO with relatively high affinity.

### Koumine Delays the Dissociation of ^3^H-PK11195 From Translocator Protein

**FIGURE 2:**
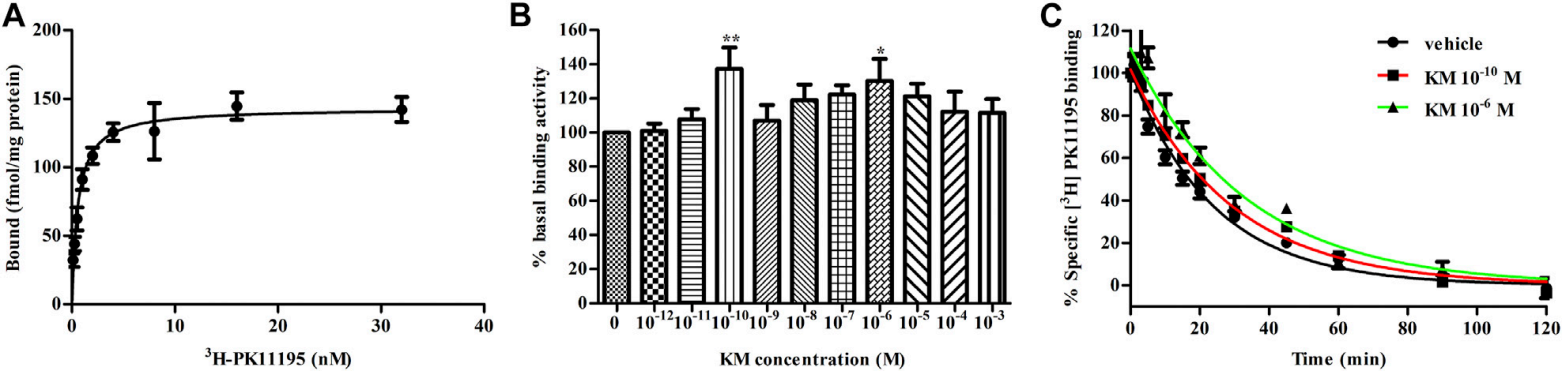
Koumine delays the dissociation of ^3^H-PK11195 from TSPO. **(A)** Saturation binding curve of specific ^3^H-PK11195 binding. **(B)** Binding activity of 0.1 nM ^3^H-PK11195 in the absence or presence of different concentrations of koumine. **(C)** The dissociation curves of ^3^H-PK11195 in the absence or presence of the indicated concentrations of koumine. For **(A)** and **(B)**, 50 *μ*g of TSPO protein was incubated for 120 min at 4°C with 0.125–32 nM ^3^H-PK11195 **(A)** or 0.1 nM PK11195 in the absence or presence of different concentrations of koumine **(B)**. Specific binding is defined as the difference between total binding and nonspecific binding in the presence of 1000-fold excess unlabeled PK11195. In **(B)**, the basal binding activity was the specific binding value (cpm) in the absence of koumine and was defined as 100%. For **(C)**, 50 *μ*g of TSPO protein was incubated for 120 min at 4°C with 10 nM ^3^H-PK11195 to achieve pre-equilibration, and dissociation was induced by 10 *μ*M PK11195 in the absence or presence of the indicated concentrations of koumine. Specific binding is defined as the difference between total binding and nonspecific binding in the presence of 10 *μ*M PK11195. Abbreviations: KM, koumine. Data are represented as the mean ± SEM of 4–5 independent experiments performed in duplicate (*n* = 4–5). *p < 0.05, **p < 0.01 vs. vehicle group. Statistical analysis in **(B)** was performed using one-way ANOVA followed by the LSD post hoc test.

As radioligand binding assays are an important tool for studying allostery, we adopt this method to assess the allosteric modulatory effects of koumine on TSPO. ^3^H-PK11195 is the only commercially available TSPO radioligand at present and was therefore chosen for use in our test. First, we examined the affinity of ^3^H-PK11195 with TSPO. The K_d_ and B_max_ values were 0.59 ± 0.10 nM and 143.3 ± 5.4 fmol/mg protein, respectively (Figure 2A), which was consistent with previous results (Benavides et al., 1983; Awad and Gavish, 1987). Then, to quantify the effects of koumine on TSPO, we conducted equilibrium binding assays with 10^−12^–10^−3^ M koumine against 0.1 nM (∼0.2 K_d_) ^3^H-PK11195. All concentrations of koumine tended to increase the binding of 0.1 nM ^3^H-PK11195. Noteworthily, the augmentation effects were not dose dependent as usual for PAMs, and the most significant augmentation effects were observed at 10^−10^ M and 10^−6^ M koumine (Figure 2B). Last, we determined the dissociation rate of ^3^H-PK11195 in the absence or presence of 10^−10^ and 10^−6^ M koumine. As shown in Figure 2C, the dissociation rates of ^3^H-PK11195 in vehicle, 10^−10^ M koumine or 10^−6^ M koumine were 0.0419 ± 0.0016, 0.0341 ± 0.0009 and 0.0301 ± 0.0024 min^−1^, respectively. Both 10^−10^ and 10^−6^ M koumine significantly inhibited the dissociation of ^3^H-PK11195 (*p* < 0.001 for 10^−10^ and 10^−6^ M koumine), indicating that koumine is a PAM with respect to ^3^H-PK11195 affinity.

### Koumine Augments Ro5-4864-Mediated Analgesic and Anti-Inflammatory Effects *in vivo*

To evaluate the potential therapeutic effects of TSPO PAM *in vivo*, several inflammatory and neuropathic pain models were selected for which Ro5-4864 has been discovered to have therapeutic effects: the formalin-induced inflammatory pain model, CIA model and CCI model of neuropathic pain.

**FIGURE 3:**
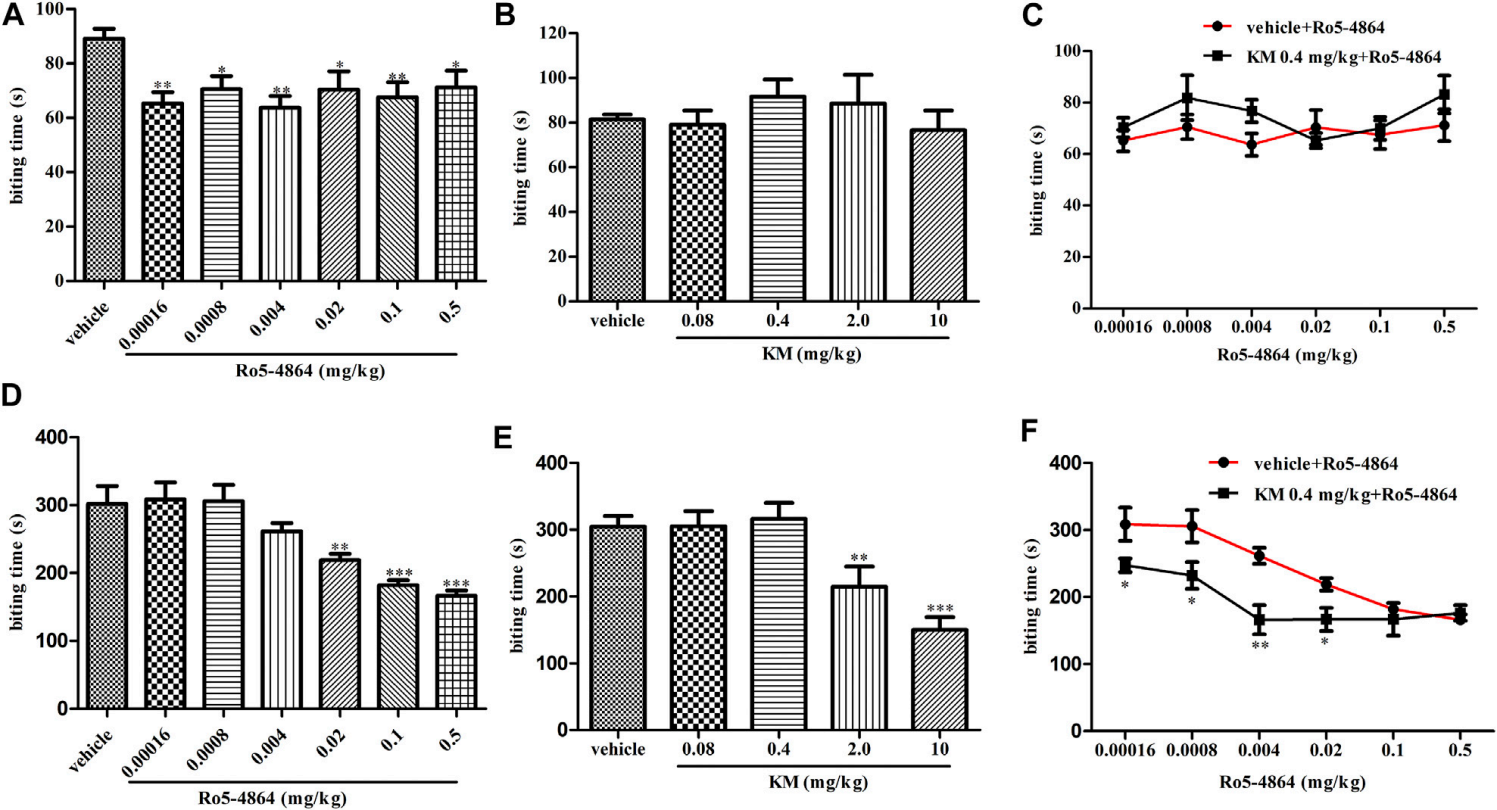
Modulation of the *in vivo* efficacy of Ro5-4864 by koumine in the formalin test in mice. **(A,B)** Dose response effects of Ro5-4864 **(A)** or koumine **(B)** on phase I in the formalin test. **(C)** Dose response curves for Ro5-4864 in the absence or presence of 0.4 mg/kg koumine. **(D,E)** Dose response effects of Ro5-4864 **(D)** or koumine **(E)** on phase II in the formalin test. **(F)** Dose response curves for Ro5-4864 in the absence or presence of an inactive dose of koumine. Koumine (0.08–10 mg/kg) or vehicle was injected s. c. 40 min before formalin injection. Ro5-4864 (0.00016–0.5 mg/kg) or vehicle was injected s. c. 30 min before formalin injection. To determine whether koumine potentiated the anti-inflammatory pain effects of Ro5-4864, koumine (0.4 mg/kg, s. c.) or vehicle was injected 10 min before Ro5-4864 (0.00016–0.5 mg/kg, s. c.) injection. Abbreviations; KM: koumine. Data are represented as the mean ± SEM, *p < 0.05, **p < 0.01, ***p < 0.001 vs. the corresponding vehicle group. Statistical analysis in **(A,B,D,E)** were performed using one-way ANOVA followed by the LSD post hoc test and in **(C,F)** were performed using the independent samples *t*-test. Each group consisted of 8–10 mice.

Consistent with its known analgesic activity (DalBo et al., 2004), in a formalin-induced inflammatory pain model, Ro5-4864 produced a dose-dependent antinociceptive effect in phase two (Figure 3D). Under similar conditions, koumine significantly inhibited the second-phase nociceptive response at 2.0 and 10 mg/kg but not 0.08 and 0.4 mg/kg (Figure 3E). Dose response curves of Ro5-4864 in the absence or presence of 0.4 mg/kg koumine were generated. The results showed that 0.4 mg/kg koumine is ineffective on its own but can significantly augment the 0.00016–0.02 mg/kg Ro5-4864-mediated analgesic effect (Figure 3F). In phase one, Ro5-4864 produced an antinociceptive effect, while koumine was ineffective, which is in agreement with previous findings (DalBo et al., 2004; Xu et al., 2012). Additionally, 0.4 mg/kg koumine did not increase the Ro5-4864-mediated analgesic response in the first phase of the formalin test (Figures 3A–C).

**FIGURE 4:**
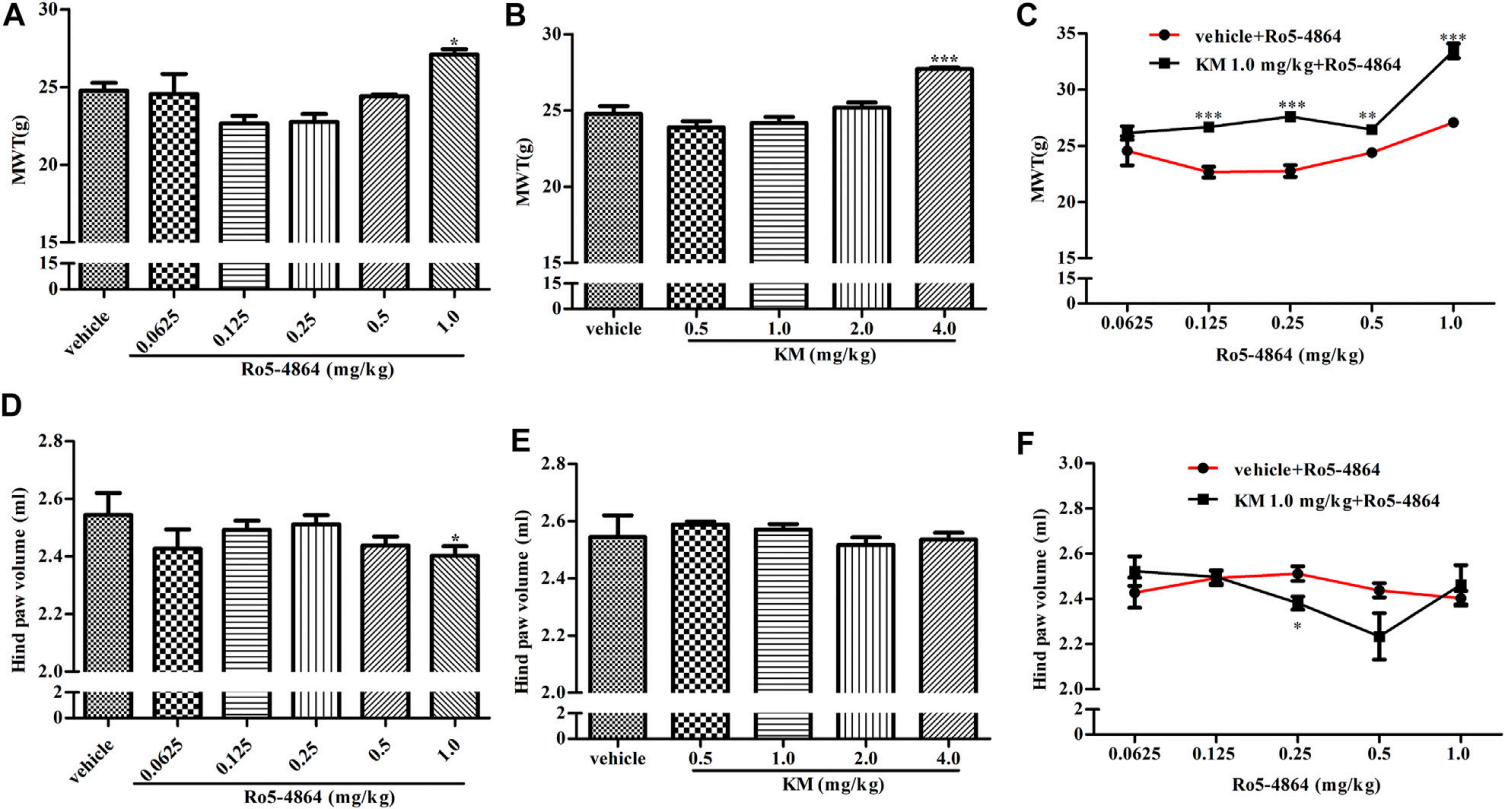
Modulation of the *in vivo* efficacy of Ro5-4864 by koumine successive administration in the rat model of CIA. **(A,B,D,E)** Effects of repeated administrations of Ro5-4864 **(A,D)** or koumine **(B,E)** on MWT **(A,B)** and hind paw volume **(D,E)**. **(C,F)** Dose response effects on the MWT **(C)** and hind paw volume **(F)** of Ro5-4864 in the absence or presence of koumine (1.0 mg/kg). Seven-week-old Lewis rats were immunized with bovine type II collagen in IFA. Immunized rats received Ro5-4864, koumine or vehicle for seven consecutive days beginning from day 29 after the first collagen injection. The MWT was determined on day 35, and the hind paw volume was measured 60 min following the MWT determination for each rat. Koumine or vehicle was injected s. c., and MWT values were determined 60 min after koumine injection. Ro5-4864 or vehicle was injected i. p., and MWT was determined 50 min after Ro5-4864 injection. To determine whether koumine potentiated the effects of Ro5-4864, koumine (1.0 mg/kg, s. c.) or vehicle was injected 10 min before Ro5-4864 (0.0625–1.0 mg/kg, i. p.) injection, and the MWT was determined 50 min after the last injection. Abbreviations; KM: koumine, MWT: mechanical withdrawal threshold. Data are represented as the mean ± SEM, *p < 0.05, **p < 0.01, ***p < 0.001 vs. the corresponding vehicle group. Statistical analysis in **(A,B,D,E)** were performed using one-way ANOVA followed by the LSD post hoc test and in **(C,F)** were performed using the independent samples *t*-test. Each group consisted of 6–10 rats.

**FIGURE 5:**
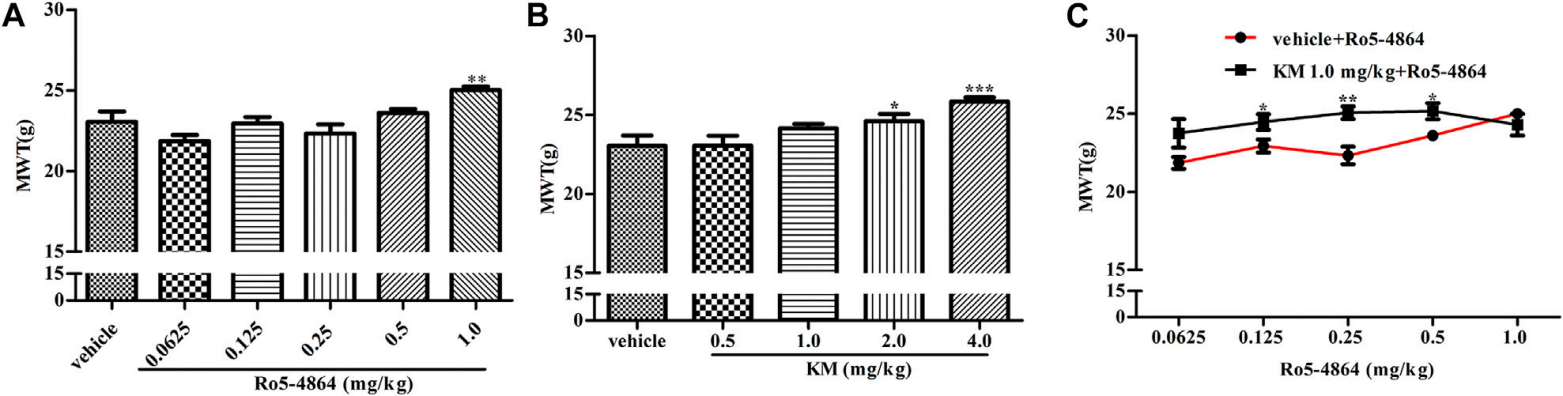
Modulation of the *in vivo* efficacy of Ro5-4864 by koumine acute administration in the rat model of CIA. **(A,B)** Effects of acute administration of Ro5-4864 **(A)** or koumine **(B)** on the MWT. **(C)** Dose response effects on the MWT of Ro5-4864 in the absence or presence of koumine (1.0 mg/kg). Seven-week-old Lewis rats were immunized with bovine type II collagen in IFA. Immunized rats received Ro5-4864, koumine or vehicle on day 29 after the first collagen injection. Koumine or vehicle was injected s. c., and the MWT was determined 60 min after koumine injection. Ro5-4864 or vehicle was injected i. p., and the MWT was determined 50 min after Ro5-4864 injection. To determine whether koumine potentiated the effects of Ro5-4864, koumine (1.0 mg/kg, s. c.) or vehicle was injected 10 min before Ro5-4864 (0.0625–1.0 mg/kg, i. p.) injection, and the MWT was determined 50 min after the last injection. Abbreviations; KM: koumine, MWT: mechanical withdrawal threshold. Data are represented as the mean ± SEM, *p < 0.05, **p < 0.01, ***p < 0.001 vs. the corresponding vehicle group. Statistical analysis in **(A,B)** were performed using one-way ANOVA followed by the LSD post hoc test and in **(C)** was performed using the independent samples *t*-test. Each group consisted of 7–11 rats.

CIA model produces long-lasting mechanical allodynia and paw edema and is a typical model of inflammatory pain. The baseline of MWT and hind paw volume on day 28 after the first collagen injection in the rat model of CIA were shown in Supplementary Figure S3, and there is no difference between each groups. In a therapeutic setting, Ro5-4864 or koumine was given daily from day 29 to day 35. The MWT was determined on day 35, and hind paw volume was measured 60 min after the MWT determination. Ro5-4864 and koumine clearly reduced mechanical allodynia (Figures 4A,B). Koumine administered alone did not produce an anti-edematogenic effect, while Ro5-4864 at the highest dose tested significantly inhibited paw edema (Figures 4D,E). When an ineffective dose of koumine (1.0 mg/kg) was coadministered with Ro5-4864, the Ro5-4864-mediated analgesic and anti-edematogenic effects at 0.125–1.0 and 0.25 mg/kg, respectively, were notably increased (Figures 4C,F). The pharmacological effect of TSPO PAM in acute administration was also evaluated. As shown in Figure 5, acute administration of Ro5-4864 29 days after the first collagen injection reduced mechanical allodynia, and treatment with koumine on day 29 also showed a dose-dependent inhibition effect. Consistent with successive administration, the coadministration of 1.0 mg/kg koumine and Ro5-4864 clearly augmented the antinociceptive effect of acute administration.

**FIGURE 6:**
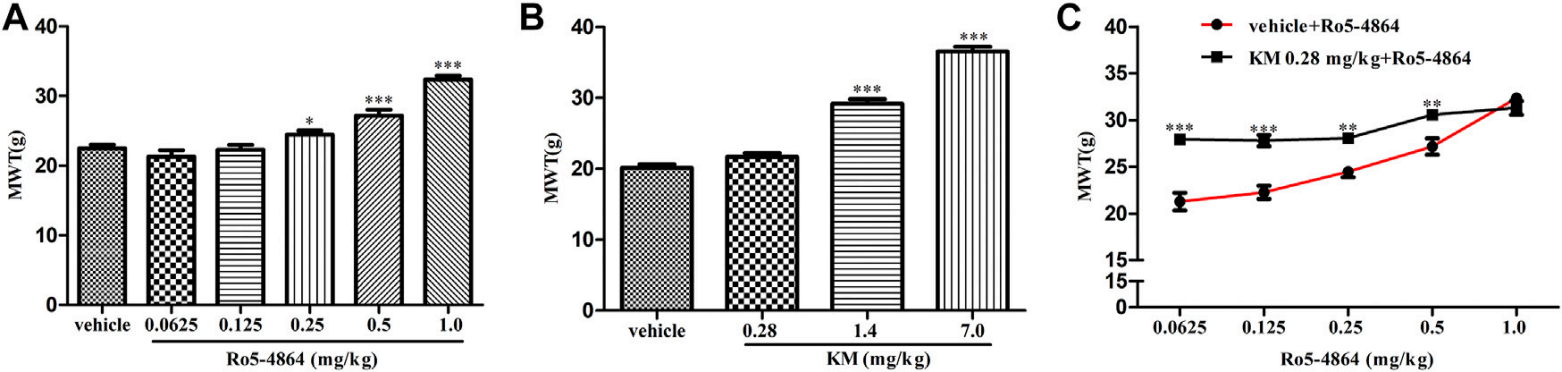
Modulation of the *in vivo* efficacy of Ro5-4864 by koumine in the rat model of CCI. **(A,B)** MWT was increased by acute Ro5-4864 **(A)** or koumine **(B)** administration in a dose-dependent manner. **(C)** Dose response effects on the MWT of Ro5-4864 in the absence or presence of koumine (0.28 mg/kg). Koumine (0.28, 1.4 and 7.0 mg/kg) or vehicle was injected s. c. 9 days after CCI surgery, the MWT was determined 60 min after koumine injection. Ro5-4864 (0.0625, 0.125, 0.25, 0.5 and 1.0 mg/kg) or vehicle was injected i. p. 9 days after CCI surgery, the MWT was determined 50 min after Ro5-4864 injection. To determine whether koumine potentiated the analgesic effects of Ro5-4864, koumine (0.28 mg/kg, s. c.) or vehicle was injected 10 min before Ro5-4864 (0.0625–1.0 mg/kg, i. p.) injection on day 9 after CCI surgery, and the MWT was determined 50 min after the last injection. Abbreviations; KM: koumine, MWT: mechanical withdrawal threshold. Data are represented as the mean ± SEM, *p < 0.05, **p < 0.01, ***p < 0.001 vs. the corresponding vehicle group. Statistical analysis in **(A,B)** were performed using one-way ANOVA followed by the LSD post hoc test and in **(C)** was performed using the independent samples *t*-test. Each group consisted of 6–10 rats.

As acute administration of Ro5-4864 and koumine was effective, the analgesic effect of acute administration of koumine and the possible positive allosteric modulatory effect were then tested in CCI-induced neuropathic pain. In the rat model of CCI, there is no difference in the baseline of MWT between each groups on day 8 after CCI surgery (Supplementary Figure S4). Treatment with Ro5-4864 9 days after surgery inhibited CCI-induced mechanical allodynia in a dose-dependent manner, consistent with previous results (Liu et al., 2016). Koumine at 1.4 and 7.0 mg/kg but not 0.28 mg/kg produced notable antinociceptive effects (Figures 6A,B). Dose response curves of the Ro5-4864-mediated analgesic effect in the absence or presence of an ineffective dose of koumine (0.28 mg/kg) were generated, and 0.28 mg/kg koumine significantly augmented 0.0625–0.5 mg/kg Ro5-4864-mediated antinociceptive activity (Figure 6C). The MWT in naïve and CCI sham-operated rats was also evaluated after Ro5-4864 or koumine administration. Neither Ro5-4864 nor koumine altered the MWT in naïve and sham-operated rats, and koumine could not significantly augment the antinociceptive effect of Ro5-4864 at the same time (Supplementary Figure S5).

### Koumine Enhances Ro5-4864- and PK11195-Mediated Anti-Proliferation Effects in T98G Human Glioblastoma Cells

Allosteric modulation can be demonstrated from multiple perspectives, and cell function experiments are an important means to demonstrate allosteric modulation. TSPO mediates cell proliferation and synthesis of steroid hormones. Therefore, in order to further demonstrate the allosteric modulatory effects of koumine, koumine was tested in two cell functional assays: inhibition of T98G human glioblastoma cell proliferation and stimulation of C6 cell pregnenolone production.

**FIGURE 7:**
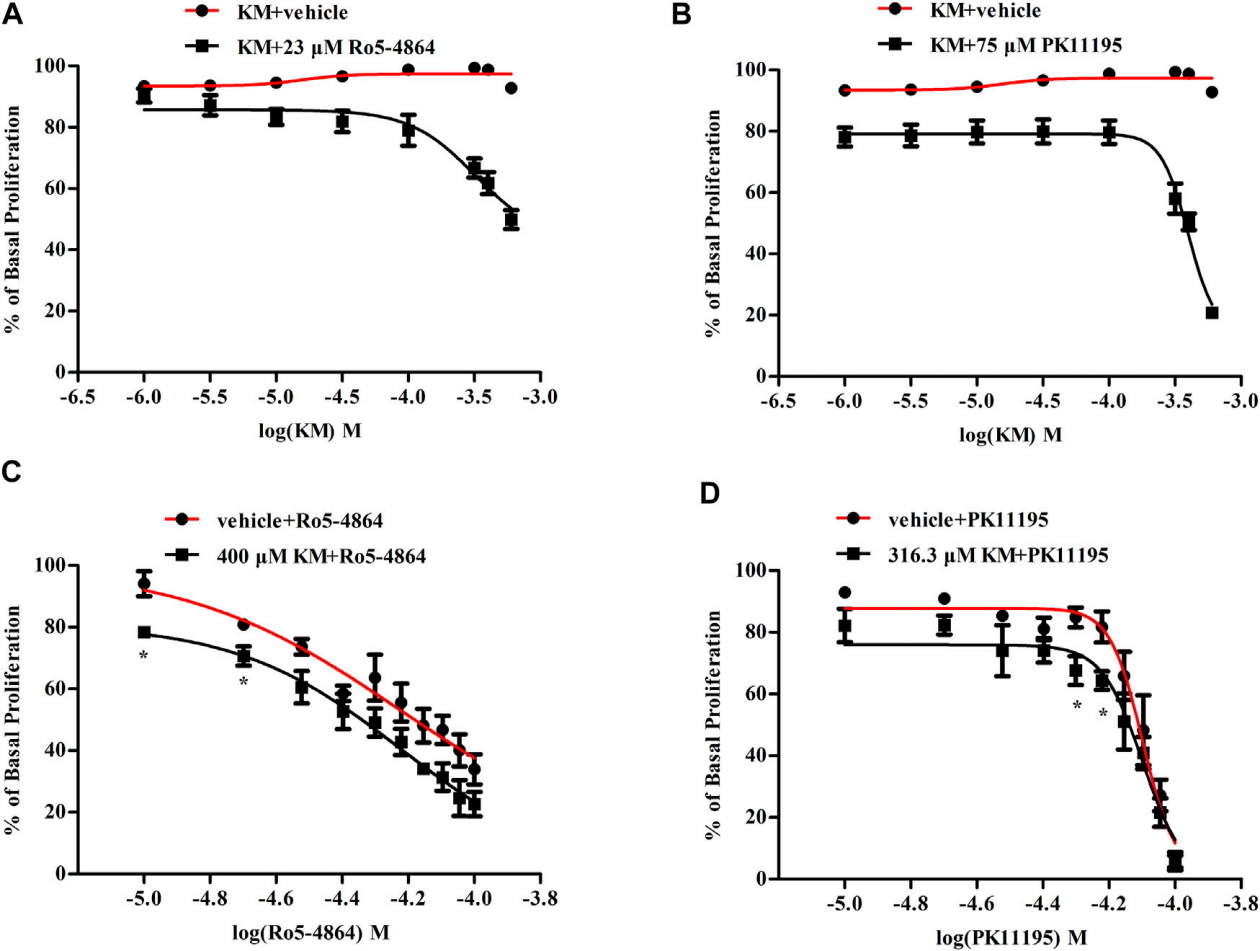
Koumine enhances Ro5-4864- and PK11195-mediated antiproliferation effects in T98G human glioblastoma cells. **(A,B)** Positive modulation of the antiproliferative effect of an EC_20_ concentration of Ro5-4864 **(A)** and PK11195 **(B)** by koumine in T98G human glioblastoma cells. **(C,D)** Dose response curve of the antiproliferative effect of Ro5-4864 **(C)** or PK11195 **(D)** in the absence or presence of 400 *μ*M or 316.3 *μ*M koumine, respectively. Cell proliferation was determined by the BrdU incorporation assay, which was measured at 370 nm (reference wavelength 492 nm). Cells treated with 0.5% DMSO alone were used as controls. Abbreviations: KM, koumine. The values are expressed as the mean ± SEM of 3–5 independent experiments performed in triplicate (*n* = 3–5), *p < 0.05 vs. the corresponding vehicle group.

In T98G human glioblastoma cells, Ro5-4864 and PK11195 dose-dependently inhibited cell proliferation with EC_50_ values of 66 and 81 *μ*M (Figures 7C,D), respectively. Koumine did not affect T98G human glioblastoma cell proliferation on its own at 1–600 *μ*M, but it significantly increased the inhibition of proliferation produced by an EC_20_ concentration of Ro5-4864 (∼23 *μ*M) and PK11195 (∼75 *μ*M) (Figures 7A,B). Concentration-response curves for Ro5-4864 and PK11195-mediated T98G human glioblastoma cell proliferation were also generated in the absence or presence of koumine (Figures 7C,D). Koumine produced a leftward shift in the potency of Ro5-4864 and PK11195, and treatment with 400 *μ*M or 316.3 *μ*M koumine augmented the Ro5-4864- and PK11195-mediated inhibition of proliferation. Cell viability was not significantly affected by koumine, Ro5-4864 or PK11195 in the same dosing regimen as proliferation (Supplementary Figure S6).

Then, the possible positive allosteric modulatory effect of koumine was measured in terms of pregnenolone production in C6 glioma cells. In this cell line, Ro5-4864 and PK11195 produced an increase of approximately 50% in pregnenolone production with EC_50_ values of 29 and 69 *μ*M, respectively (Supplementary Figures S7A,B). Koumine used alone also exhibited some agonist activity with an EC_50_ of 270 mM and E_max_ of 33% (Supplementary Figure S7C). In addition, koumine did not significantly increase the pregnenolone production activity produced by an EC_20_ concentration of Ro5-4864 (∼11 *μ*M) and PK11195 (∼27 *μ*M) (Supplementary Figures S7D,E). The divergence between the PAM activities of koumine seen in the antiproliferation effect in T98G human glioblastoma cells and the lack of PAM activity seen in this assay may be attributable to differences in receptor reserve between the two assays and/or cell lines. Functional selectivity of an allosteric modulator may be another possible explanation for this observation, as an allosteric modulator may not uniformly activate its various cellular signaling pathways linked to a given receptor (Kenakin and Christopoulos, 2013).

## Discussion

In this study, we report the pharmacological characterization of koumine as a TSPO PAM. Koumine might be a high-affinity ligand of TSPO and that it likely acts as a PAM since it could delay the dissociation of ^3^H-PK11195 from TSPO. Importantly, koumine augments Ro5-4864-mediated analgesic and anti-inflammatory effects in several acute and chronic inflammatory and neuropathic pain models. Furthermore, in T98G human glioblastoma cells, koumine enhancs Ro5-4864- and PK11195-mediated antiproliferation effects. Together, we provide compelling evidence that allostery may occur in the interaction of TSPO and TSPO PAM, which may provide a promising novel avenue for the discovery of analgesics. TSPO is the target of koumine, which exerts its pharmacological effects through the positive allosteric modulation of TSPO.

*Gelsemium elegans* Benth*.* has been used in Chinese traditional medicine for the treatment of pain, and a previous preclinical study reported that crude *Gelsemium* alkaloid extract has a significant analgesic effect in a variety of pain models with no morphine-like tolerance or dependence (Tan et al., 1988). Further clinical evidence has shown that a parenteral solution of crude *Gelsemium* alkaloid extract has potent analgesic effects, and the effective rate of analgesia was 90% (Chen, 1984). However, the narrow therapeutic index limits the further clinical use of crude alkaloid extraction. Koumine is the most abundant monomer compound in the crude alkaloid extraction of *Gelsemium elegans* Benth. with low toxicity. Our research group has previously systematically explored the pharmacodynamics, pharmacokinetics and safety of koumine and found that it has significant anti-inflammatory and neuropathic pain effects with a high therapeutic index and that the therapeutic effects may be related to attenuating neuroglia activation, where TSPO is abundantly expressed (Gui et al., 2020). It is expected to become a new clinical candidate for analgesia (Xu et al., 2012; Ling et al., 2014; Yang et al., 2016; Xiong et al., 2017; Jin et al., 2018a; Jin et al., 2018b; Jin et al., 2019; Luo et al., 2020; Su et al., 2020; Ye et al., 2020).

A characteristic of allosteric modulators is their ability to bind receptors and modulate the binding and/or signaling efficacy of orthosteric ligands (Wootten et al., 2013). SPR, a gold standard for real-time and label-free measurements of biomolecular interactions included in the pharmacopoeias of several countries, measuring affinity is its main application (Kameyama et al., 2019). Since there is no isotope labeling of the koumine, the traditional radioligand binding experiment cannot be used to determine the affinity. Therefore, we used the SPR technique to determine the affinity of TSPO and koumine. The results showed that the affinities of Ro5-4864, PK11195 and protoporphyrin IX obtained by SPR technology and radioligand binding experiment results from literatures (the K_D_ values of Ro5-4864, PK11195 and protoporphyrin IX were 7.3 ± 0.8, 0.63 ± 0.06 and 14.5 ± 10.7 nM, respectively (Awad and Gavish, 1987; Verma et al., 1987)) are in the same order of magnitude, and their changing trends are consistent, which indicates that SPR technology can be used for TSPO ligand affinity research. As for the slightly difference of PK11195 affinity between our SPR and radioligand binding experiments, this may be mainly related to the different experimental system and sample sources (in SPR and radioligand binding experiments, rat cerebral cortex TSPO and prokaryotically expressed TSPO protein were used, respectively). Here, the reliable SPR results showed that the K_D_ of koumine was 0.86 ± 0.07 nM, which was significantly lower than the values of Ro5-4864, PK11195 or protoporphyrin IX (*p* < 0.05 for Ro5-4864 and PK11195, *p* < 0.001 for protoporphyrin IX), indicating that koumine can bind TSPO directly and is a high-affinity ligand of TSPO. Thus, TSPO may be a therapeutic target of koumine.

Allostery can be studied at the molecular level, cell function level and overall animal effect level, and radioligand binding assays are vital tools for studying allostery at molecular level, as they can often validate allostery directly (Christopoulos et al., 2014). A classic feature of a PAM is the ability to inhibit the dissociation rate of orthosteric ligands (Wootten et al., 2013). In this study, our results indicated that koumine can delay the dissociation of ^3^H-PK11195 from TSPO, acting as a PAM with respect to ^3^H-PK11195 affinity. In the presence of 10^−10^ M and 10^−6^ M koumine, the dissociation rate of ^3^H-PK11195 was 81 and 72% of the dissociation rate of the vehicle group, respectively, and the inhibition intensity is similar to that of other research group, which was 67% of vehicle group (Guo et al., 2013). The residence time (1/K_off_) of ^3^H-PK11195 in vehicle, 10^−10^ M koumine or 10^−6^ M koumine were 23.9, 29.3 and 33.2 min, respectively. Noteworthily, in the equilibrium binding assay, koumine increased the binding of 0.1 nM ^3^H-PK11195 in a non-dose-dependent manner, and only 10^−10^ M and 10^−6^ M koumine significantly increased the binding. One possible explanation for this observation is that TSPO forms a complex with voltage-dependent anion channel (VDAC) and adenine nucleotide transporter (ANT) (McEnery et al., 1992), and the TSPO/VDAC/ANT complex may complicate the binding profile between koumine and PK11195 (Shoshan-Barmatz et al., 2019; Lee et al., 2020). Such complicated binding properties have also appeared in the allosteric modulation of other protein complexes, and it exhibits a biphasic effect rather than a dose-dependence (Bonaventura et al., 2015). In addition, the rat cerebral cortex was used in the radioligand binding experiment on our manuscript. Although it can well reflect the actual situation *in vivo*, TSPO proteoliposomes can be used for further studies in order to more directly study the influence of koumine on the affinity between TSPO and ^3^H-PK11195.

Whether allostery has therapeutic effects *in vivo* is an important question to resolve, subsequently, we studied the allosteric modulatory effects of koumine on TSPO from the overall animal effect level. The formalin-induced inflammatory pain model, CIA model and CCI model are typical pain models that can simulate the clinical symptoms of acute and chronic inflammatory pain and neuropathic pain, respectively, and are currently widely used in the screening of analgesics (Ashraf et al., 2016; Takasaki et al., 2019; Xia et al., 2019). Therefore, these classic inflammatory and neuropathic pain models were chosen. Considering that Ro5-4864, rather than PK11195, has been discovered to have therapeutic effects in neuropathic pain models (Liu et al., 2016), the therapeutic effect of an ineffective dose of koumine combined with Ro5-4864 was explored. It is worth noting that when an ineffective dose of koumine was combined with Ro5-4864, it significantly augmented Ro5-4864-mediated analgesic and anti-inflammatory effects in all the inflammatory and neuropathic pain models we tested. These results further proving allostery in TSPO and providing the first proof of principle that TSPO PAM may provide a novel avenue for the discovery of analgesics.

Allosteric modulators generally include PAM, negative allosteric modulators, and neutral allosteric ligands. In addition, some allosteric modulators exhibit PAM effects at low concentrations, and can also exert their effects alone at high concentrations, showing allosteric agonist effects, these allosteric modulators are referred to as ago-PAM (Schwartz and Holst, 2007; Garai et al., 2018). In this study, in addition to augmenting Ro5-4864-mediated effects *in vivo*, koumine alone significantly inhibited the nociceptive response at high dose we used, which was in agreement with our previous findings (Xu et al., 2012; Ling et al., 2014; Yang et al., 2016; Xiong et al., 2017; Jin et al., 2018a; Jin et al., 2018b; Jin et al., 2019). Therefore, we speculated that koumine is the ago-PAM of TSPO. Moreover, our results also shows that the potent analgesic koumine may have a different action mechanism from currently used analgesics, that is, positive allosteric modulation of TSPO. Compared with conventional orthosteric ligand, allosteric modulator has many advantages, such as high selectivity and strong safety profile. It has been an emerging paradigms in drug development and several efficient allosteric modulators has approved as marketed drugs (Wootten et al., 2013; Cheng and Jiang, 2019). Thus, koumine, the TSPO PAM, may be a valuable clinical candidate.

A definitive evidence of an allosteric modulator is structure determination to show that its binding site is spatially distinct from and nonoverlapping with the orthosteric sites (Christopoulos et al., 2014). The specific binding sites of TSPO and its ligands have not been fully elucidated. The current research results show that PK11195 and Ro5-4864 bind to the N-terminus of TSPO, and PK11195 competes with the endogenous ligand (such as protoporphyrin IX) of TSPO (Jaremko et al., 2015). In the radioligand binding experiments in this article, compared with the competition of traditional TSPO orthosteric ligand with ^3^H-PK11195, koumine can significantly increase the binding of ^3^H-PK11195 to TSPO, indicating that the binding site of koumine and TSPO was different from that of the traditional orthosteric ligand, that is, koumine was bound to the allosteric binding site. However, the specific binding site of TSPO bound to koumine has yet to be confirmed by future structure determination. Interestingly, PK1195 and koumine did not produce competitive effects in the radioligand binding experiments. However, in the postoperative pain model of rats, our previous study found that intrathecal injection of 0.7 *μ*g PK11195 had no analgesic effect, but it weakened the analgesic effect of intrathecal administration of 200 *μ*g koumine, and 7 *μ*g PK11195 alone can also produce a certain analgesic effect (Xiong et al., 2017). The analgesic effect of PK11195 has also been reported in formalin-induced inflammatory pain models (DalBo et al., 2004). We speculate that in the postoperative pain model, low dose PK11195 has no analgesic effect, but as the dose increases, it can also produce a certain analgesic effect. Moreover, in addition to exerting analgesic effects as an allosteric agonist, high dose koumine may also exert partial analgesic effects through positive allosteric modulate endogenous ligands (such as protoporphyrin IX). When low dose PK11195 is used in combination with koumine, the binding of PK11195 to the orthosteric site of TSPO reduces the binding of endogenous ligands, thereby weakening the analgesic effect of koumine.

The foregoing studies fully characterized the allosteric modulatory effect of koumine on TSPO from the molecular level and the overall animal effect level, indicating that koumine is a PAM of TSPO. In addition, studying allosteric modulation from the level of cell function is also an important method (Christopoulos et al., 2014). TSPO is involved in cell proliferation and steroid hormone synthesis, and we therefore adopted the classic PAM research protocol to further explored TSPO PAM activity in these two cell functional assays, in which the combination of koumine and EC_20_ concentration of PK11195/Ro5-4864 is applied to observe whether it can enhance the cellular functional effect of EC_20_ concentration of PK11195/Ro5-4864, furthermore, the effect of koumine on the concentration-response curves of PK11195/Ro5-4864 is explored (Burford et al., 2013). In T98G human glioblastoma cells, koumine produced a leftward shift in the potency of Ro5-4864 and PK11195, moreover, koumine did not affect T98G human glioblastoma cell proliferation alone at 1–600 *μ*M but significantly increased the inhibition of proliferation produced by EC_20_ concentrations of Ro5-4864 and PK11195, functioning as a PAM of Ro5-4864 and PK11195 efficacy for the inhibition of cell proliferation. This result further proves the allosteric modulatory effect of koumine on TSPO at the cellular level. Functional selectivity is a typical feature of allosteric modulators, that is, for a given receptor, allosteric modulator may not uniformly activate its various cellular signaling pathways. Here, the allostery was not observed in steroidogenesis, the functional selectivity of an allosteric modulator may account for the divergence between the PAM activities of koumine in these two cell functional assays. Moreover, the differences in receptor reserve between the two assays and/or cell lines may be another possible explanation for this observation (Kenakin and Christopoulos, 2013).

In conclusion, we have identified and characterized koumine as a TSPO PAM, and it can greatly enhance Ro5-4864-mediated analgesic and anti-inflammatory effects. Our data helps to deepen the understanding of koumine's therapeutic target and molecular mechanism, and lays a solid foundation for the use of the clinical candidate koumine to treat inflammatory and neuropathic pain. These results indicate that koumine, as PAM of TSPO, has great value in the development of new drugs. At the same time, our results further demonstrate the allostery in TSPO, and provide the first proof of principle that TSPO PAM may be a novel avenue for the discovery of analgesics.

## Data Availability

The original contributions presented in the study are included in the article/Supplementary Material, further inquiries can be directed to the corresponding authors.
