# Supplementary material for: Identification of Koumine as a Translocator Protein 18 kDa Positive Allosteric Modulator for the Treatment of Inflammatory and Neuropathic Pain
Source: Front Pharmacol. 2021 Jun 24;12:692917. doi: 10.3389/fphar.2021.692917 (PMC8264504; doi:10.3389/fphar.2021.692917)
Supplement: Supplementary file 1 [file DataSheet1.PDF]

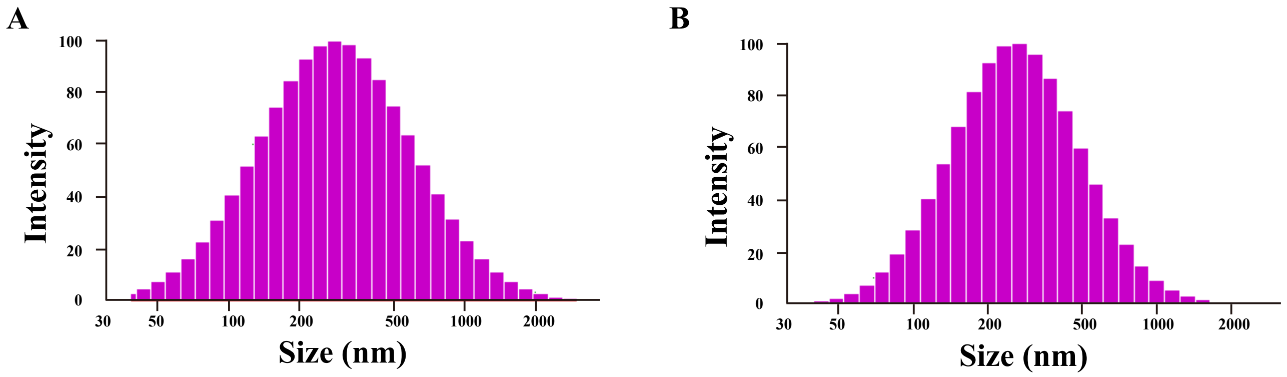

Figure S1 Representative size distribution image of liposomes (A) and proteoliposomes (B).

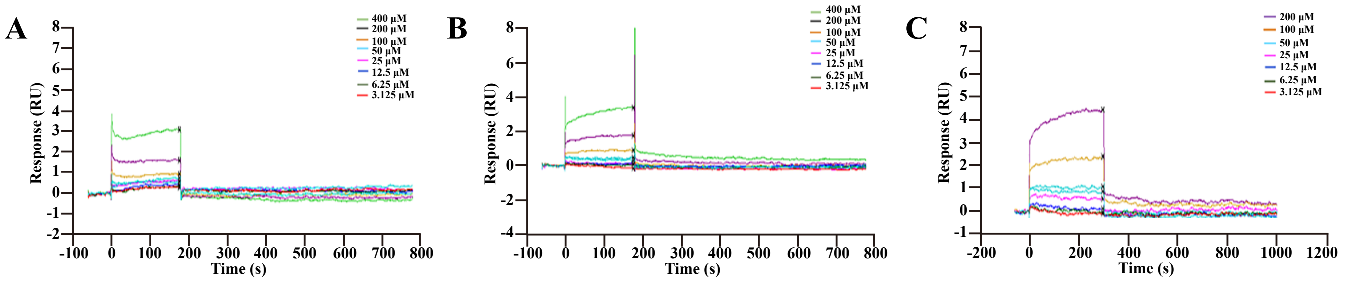

Figure S2 Kinetic profile for Ro5-4864, PK11195 and koumine binding to TSPO determined using multicycle kinetics. (A-C) The sensorgram of Ro5-4864 (A), PK11195 (B) and koumine (C) binding to the TSPO. Sensorgrams show blank and reference subtracted data, and a DMSO correction was also applied. A representative result from three independent experiments is presented.

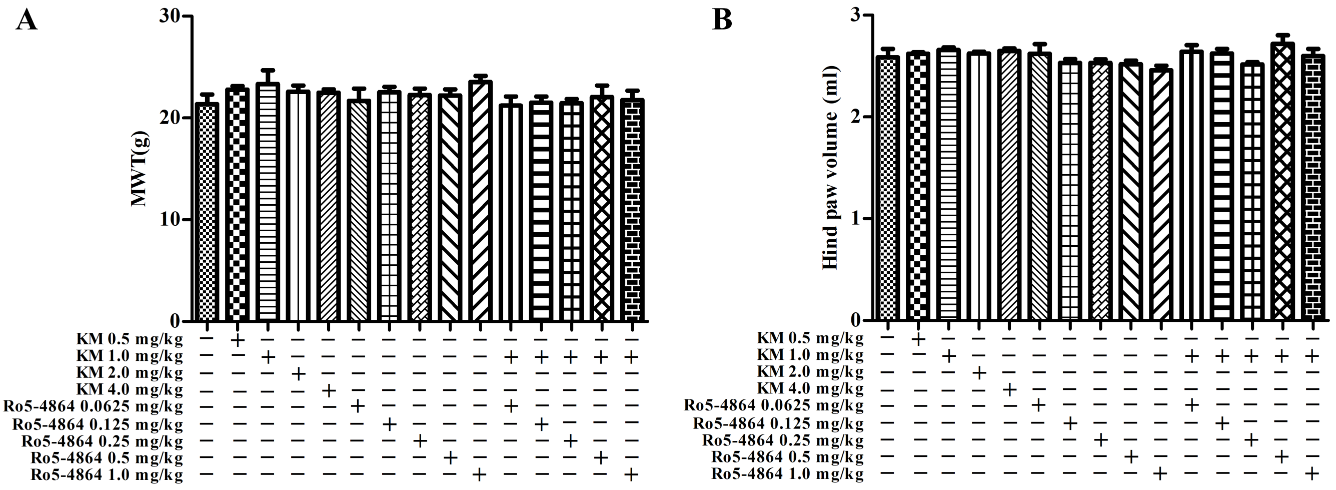

Figure S3 The baseline of MWT and hind paw volume on day 28 after the first collagen injection in the rat model of CIA. (A and B): the baseline of MWT (A) and hind paw volume (B) on day 28 in the rat model of CIA. Seven weeks old Lewis rats were immunized with bovine type II collagen in IFA, and the MWT and hind paw volume were determined on day 28 after the first collagen injection. Abbreviations; KM: koumine, MWT: mechanical withdrawal threshold. Data are represented as mean ± SEM, statistical analysis was performed using one-way ANOVA followed by the LSD post hoc test. Each group consisted of 6–10 rats.

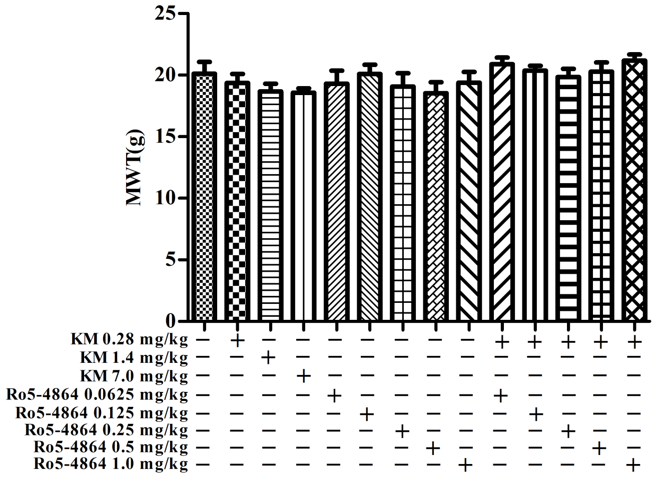

Figure S4 The baseline of MWT on day 8 after CCI surgery in the rat model of CCI. Abbreviations; KM: koumine, MWT: mechanical withdrawal threshold. Data are represented as mean ± SEM, statistical analysis was performed using one-way ANOVA followed by the LSD post hoc test. Each group consisted of 7–11 rats.

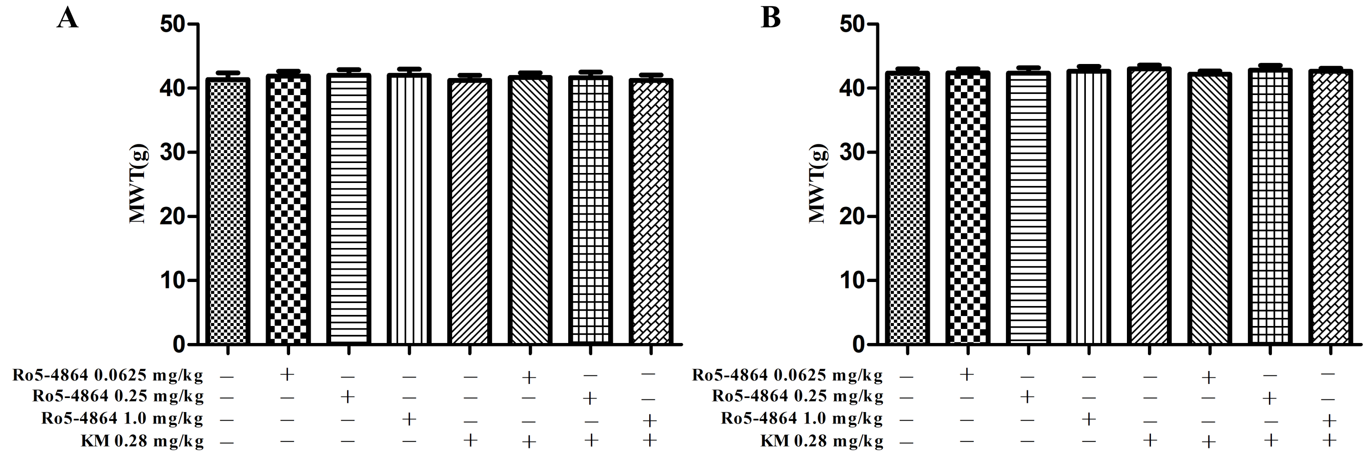

Figure S5 Modulation of the in vivo efficacy of Ro5-4864 by koumine in the naïve and CCI sham rat model. (A and B) Ro5-4864 in the absence or presence of koumine (0.28 mg/kg) had no effect on the MWT in either the naïve (A) or CCI sham (B) rat model. Koumine (0.28 mg/kg) was injected s.c. 9 days after CCI sham surgery, the MWT was determined 60 min after koumine injection. Ro5-4864 (0.0625, 0.25 and 1.0 mg/kg) was injected i.p. 9 days after CCI sham surgery, the MWT was determined 50 min after Ro5-4864 injection. To determine whether koumine potentiated the analgesic effects of Ro5-4864, koumine (0.28 mg/kg, s.c.) was injected 10 min before Ro5-4864 (0.0625-1.0 mg/kg, i.p.) injection on day 9 after CCI sham surgery, and the MWT was determined 50 min after the last injection. Abbreviations; KM: koumine, MWT: mechanical withdrawal threshold. Data are represented as the mean ± SEM, statistical analysis was performed using the independent samples t-test and when appropriate with one-way ANOVA followed by the LSD post hoc test. Each group consisted of 6–8 rats.

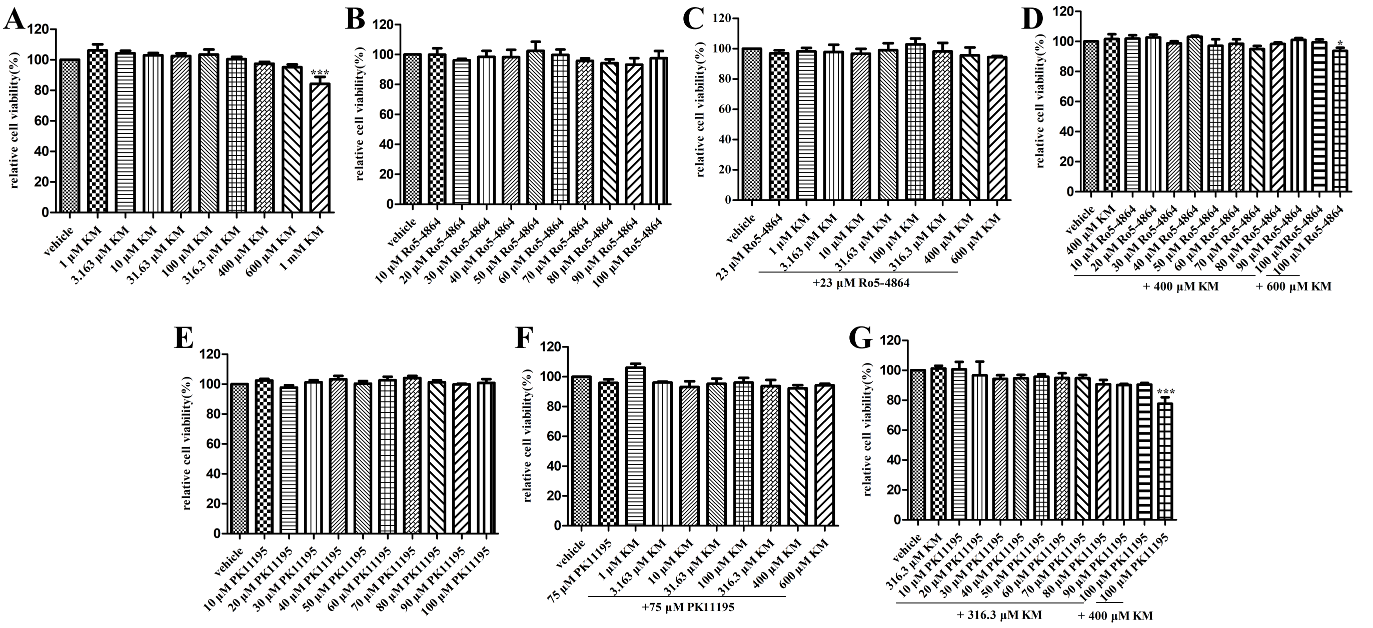

Figure S6 Effects of koumine, Ro5-4864 and PK11195 on the cell viability of T98G human glioblastoma cells. (A, C and F) Cell viability of koumine on T98G human glioblastoma cells in the absence (A) or presence of an EC_{20} concentration of Ro5-4864 (C) or PK11195 (F). (B, D, E and G) Dose response effects on cell viability of Ro5-4864 (B, D) and PK11195 (E, G) in the absence or presence of koumine. Cells were treated with various concentrations of drugs as indicated for 48 h, and cell viability was determined by the CellTiter blue cell viability assay, measured at 570 nm (reference wavelength 600 nm). Cells treated with 0.5% DMSO alone were used as the vehicle. Abbreviations: KM, koumine. The values are expressed as the mean ± SEM of 3-5 independent experiments performed in triplicate (n =3-5), **p* < 0.05, ****p* < 0.001 versus the corresponding vehicle group. Statistical analysis was performed using the one-way ANOVA followed by the LSD post hoc test.

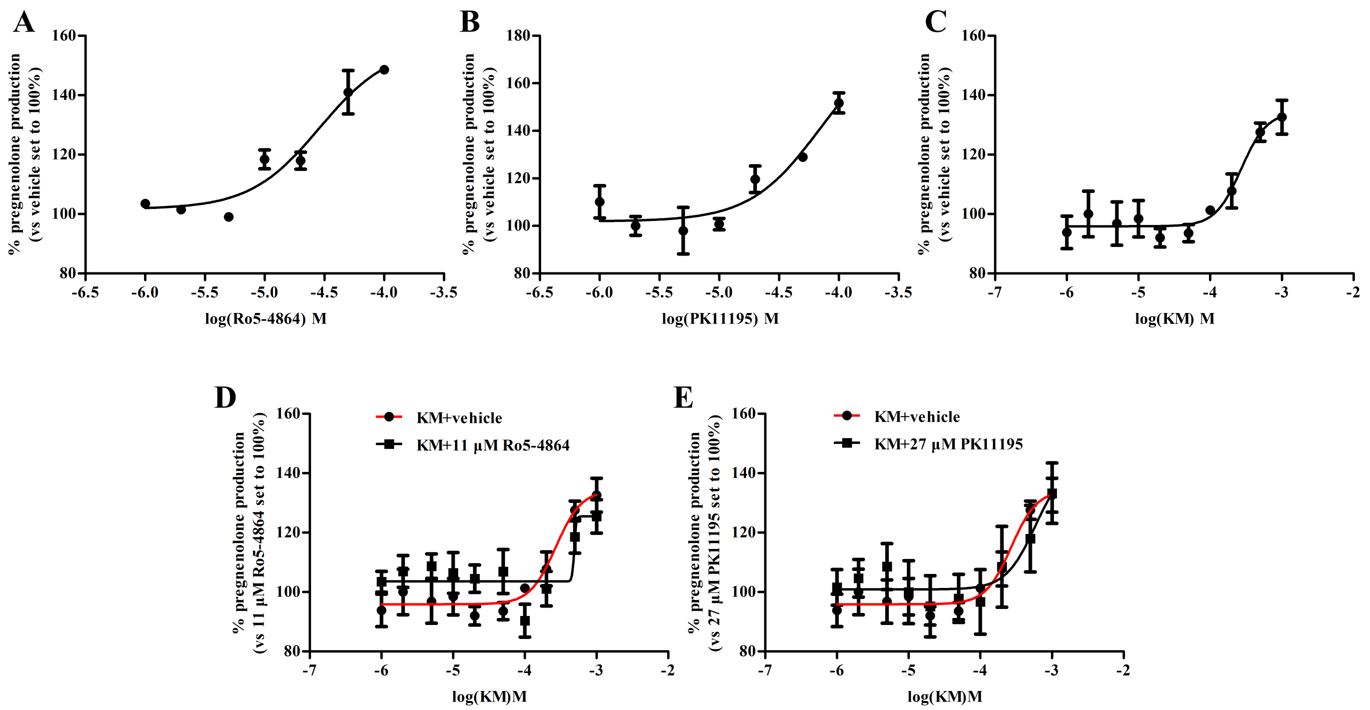

Figure S7 Effects of koumine on the pregnenolone production activity of Ro5-4864 or PK11195 in C6 glioma cells. (A-C) Dose response curve of the pregnenolone production of Ro5-4864 (A), PK11195 (B) or koumine (C) alone in C6 glioma cells. (D and E) The effects of koumine on pregnenolone production at EC_{20} concentrations of Ro5-4864 (D) and PK11195 (E). Cells were treated with various concentrations of drugs as indicated for 2 h in serum-free medium, and the pregnenolone released into the medium was assessed by ELISA, measured at 450 nm. For A-C, 100% pregnenolone production represents vehicle (0.5% DMSO) production. For D and E, 100% pregnenolone production is normalized to pregnenolone production at an ~EC_{20} concentration of Ro5-4864 (D) or PK11195 (E). Abbreviations: KM, koumine. The values are expressed as the mean ± SEM of 3-5 independent experiments performed in triplicate.
